# The effect of fibroblast growth factor 2 on neovascular vessels depends on the stage of angiogenesis

**DOI:** 10.1016/j.heliyon.2024.e39843

**Published:** 2024-10-25

**Authors:** Yuki Hattori, Haruhiko Yamada, Hidetsugu Mori, Shinpei Oba, Kaito Yokota, Masatoshi Omi, Yuichi Yamamoto, Keiko Toyama, Masayuki Ohnaka, Kanji Takahashi, Hisanori Imai

**Affiliations:** a Department of Ophthalmology, Kansai Medical University, Osaka, Japan; b Yamada Eye Clinic, Osaka, Japan

**Keywords:** FGF2, Vessels maturation, OCTA, Apelin, Ang1, PDGFRβ, Intravitreal *anti*-VEGF treatment

## Abstract

**Objective:**

The exact relationship between fibroblast growth factor 2 (FGF2) and choroidal neovascularization (CNV) remains unclear. In this study, using optical coherence tomography angiography (OCTA) and FGF2-tg mice which are transgenic mice with a rhodopsin promoter/FGF2 gene fusion, we aimed to investigate the dynamics of FGF2's role in angiogenesis over time.

**Methods:**

We developed laser-induced CNV models of FGF2-tg and wild-type (WT) mice and then separated them into two groups using different laser photocoagulation (PC) conditions. The first group received 3 intense PC shots (1st PC) altogether (**one-time PC group**), while the other group received 3 intense PC shots (1st PC) followed by 6 additional weak PC shots (2 nd PC) on the 7th day after 1st PC (**two-times PC group**).

**Results:**

Using OCTA to observe vessel changes within the same individual over time, there was no difference in the timing of vessel transition from the CNV development phase to the CNV regression phase between FGF2-tg and WT mice in the one-time PC group. In contrast, the neovascular vessels in the two-times PC group of FGF2-tg mice were maintained at least 28 days post-2nd PC without regression. In addition, mature vessels surrounded by PDGFRβ positive pericytes and α-SMA positive smooth muscle cells were observed. Real-time qPCR showed a substantial increase in apelin mRNA expression in the one-time PC group of FGF2-tg, rather than VEGF-A (p < 0.05, n = 5 or 6). Moreover, the expression levels of PDGFRβ, apelin, and Ang1 were significantly higher in FGF2-tg mice of two-times PC group than in WT mice (p < 0.05, n = 5 or 6).

**Conclusions:**

FGF2 not only promotes neovascularization via the apelin/APJ system, which is independent of VEGF signaling pathway, but also helps maintain and stabilize pre-existing neovascular vessels by stimulating PDGFRβ and Ang1. The effect of FGF2 on the neovascular vessels depends on the stage of angiogenesis.

## 1 Introduction

Angiogenesis is a complex biological process initiated by the local production of angiogenic growth factors, including angiopoietin, fibroblast growth factor (FGF), platelet-derived growth factor (PDGF), tumor necrosis factor-α (TNF-α), and vascular endothelial growth factor (VEGF), as well as the upregulation of their receptors in response to ischemia, which is mainly mediated by hypoxia-inducible factor-α (HIF1α) [1]. Furthermore, angiogenesis involves the proliferation, migration, and sealing of broken vascular endothelial cell (EC) junctions through intercellular connections, followed by the stabilization of neovascular vessels by adhesion to mural cells (MC) [2]. During this process, proangiogenic factors such as VEGF-A and FGF2 regulate defined subpopulations of ECs, promoting migration at unique tip cells and proliferation of stalk cells [3]. MCs are also recruited to stabilize new blood vessels by stimulating PDGF and TGF-β1. Following this process, ECs stop migrating and proliferating during maturation and restore the barrier function of vessels. Therefore, it is assumed that distinct signaling pathways are required to stabilize vessels, which differ from those that ECs mobilization. Among these angiogenic growth factors, FGF2 has a potent angiogenic activity and can induce new blood vessel formation in vivo [4]. Additionally, FGF2 indirectly promotes new vessel formation by controlling other angiogenic growth factor systems that play more direct roles in specific angiogenic processes. For instance, FGF2 stimulates the production of angiogenic factors such as VEGF-A and apelin, thereby inducing pathological proliferation and migration of vascular ECs. Additionally, it contributes to vessel maturation and stabilization by stimulating PDGF, angiopoietin-1 (Ang1), and vascular endothelial-cadherin (VE-cadherin) [4]. Interestingly, VEGF-A promotes vascular growth and has been found to increase vascular permeability, which can compromise the barrier function while its effects last. In contrast, FGF2 does not induce vascular hyperpermeability. The capillaries promoted by FGF2 are tightly sealed and they are morphologically different from VEGF-A-induced capillaries [[5], [6], [7]]. The difference in capillary nature may provide a hint to solve the enigma of vascular mutualization in chronic wet age-related macular degeneration (AMD).

Although FGF2 plays a crucial role in all vascular growth, the exact relationship between FGF2 and CNV remains unclear. One of the barriers to research in this field is the lack of investigation into time-course changes in the same individual. Fortunately, we have optical coherence tomography angiography (OCTA) for animals, which allows non-invasive observation of blood vessel changes over time in the same individual without invasive dye injection or animal sacrifice. Moreover, we have rhodopsin-promoted fibroblast growth factor 2 transgenic (FGF2-tg) mice. Unlike most other growth factors, FGF2 lacks a secretory signal peptide and is a cytosolic protein that exerts its effects only when FGF2 is released into the extracellular lumen [8]. Taking this feature, FGF-tg mice can simultaneously cause neovascularization and increase the expression of FGF2 in single-laser photocoagulation. In fact, in accordance with Yamada et al. [8], extensive neovascularization was observed after laser photocoagulation in our study. Therefore, we believe that the FGF2-tg mouse is a highly beneficial model for validating the physiological effects of FGF2 in the retina.

In this study, using OCTA and FGF2-tg mice, we evaluated vessel changes induced by FGF2 over time and compared these changes in FGF2-tg mice as well as wild-type (WT) mice. Furthermore, we conducted histological and biochemical analyses to better understand the role of FGF2 in neovascularization, vessel maintenance, and stabilization. Based on these results, we aimed to investigate the dynamics of FGF2's role in angiogenesis over time and propose innovative therapeutic strategies.

## 2 Materials and methods

### 2.1 Animals

**Fig. 1:**
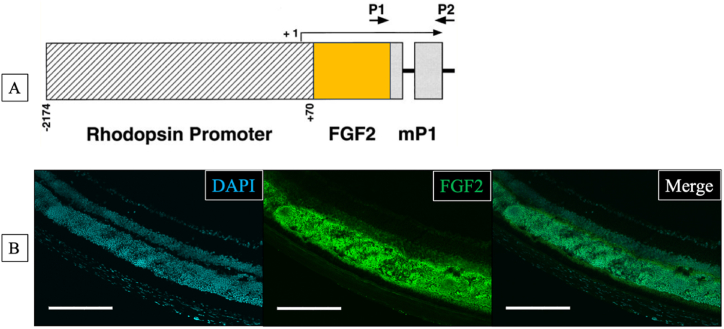
Schematic map of the rhodopsin/FGF2 fusion gene [9] and immunolabeled imaging of the retinal frozen section of FGF2-tg. **A:** Schematic map of the rhodopsin/FGF2 fusion gene. The bovine rhodopsin promoter is the previously described −2174 to +70 fragment. The FGF2 fragment extends from +84 to +1018 of the FGF2 gene with translation start sites at +280, +322, and +445 and the stop codon at +910 to +912. The oligonucleotide primers used to screen genomic DNA for the presence of the transgene are shown as P1 and P2. The primers used for RT-PCR are shown as P3 and P2. The transcription start site is indicated by +1 [9]. **B:** A frozen section of FGF2-tg mouse immunolabeled with anti-human FGF2 antibody (center). The left slide shows nuclei immunolabeled with DAPI from the same specimen. The right slide shows a merged view of the left two slides, showing that FGF2 is mainly localized in the outer nuclear layer of the retina. Scale bar = 200 μm.

We used 6–8-week-old FGF2-tg (C57bl/6J) and wild-type (WT: FGF2 negative mice of a litter) mice. FGF2-tg mice, rhodopsin-promoted fibroblast growth factor 2 transgenic mice model (Fig. 1), were procured from Peter A. Campochiaro, Wilmer Eye Institute, Johns Hopkins University [10]. All animal experiments followed the guidelines of the ARVO Statement for the Use of Animals in Ophthalmic and Vision Research and were approved by the Animal Care Committee of Kansai Medical University (approval number: 24–030). All mice were housed in pathogen-free plastic cages with 12 h light/dark cycles and had continuous free access to water and food. All plastic cages, water and bedding feed were purified before use. Anesthetization was induced by an intraperitoneal injection of 90 mg/kg ketamine hydrochloride (Daiichi Sankyo Co., Tokyo, Japan) and 40 mg/kg xylazine (Bayer, Berlin, Germany). The pupils were dilated using topical 0.5 % tropicamide and 0.5 % phenylephrine (Santen Pharmaceutical, Osaka, Japan).

### 2.2 Laser-induced choroidal neovascularization model (Li-CNV model)

CNV was induced in the left eye of each mouse by laser photocoagulation (PC: GYC-2000; NIDEK Co., Japan; wavelength 532 nm) attached to a slit-lamp delivery system (Carl Zeiss SL 130, Jena, Germany).

**Fig. 2:**
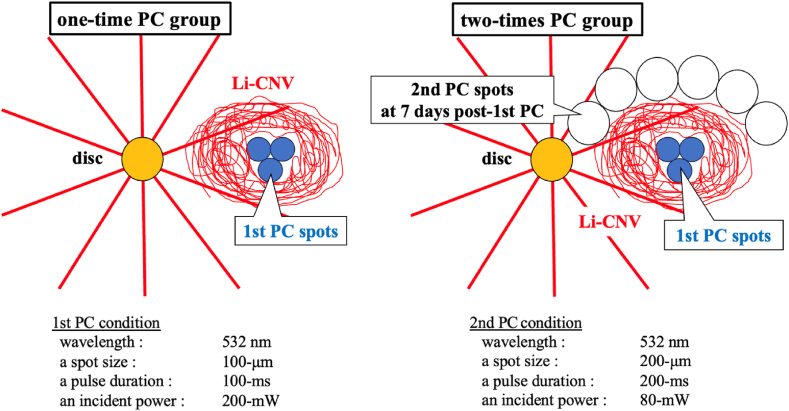
Schematic figure of Li-CNV models, Left figure: One-time PC group mice receive 3 intense PC shots altogether (1st PC, drawn as blue circles). After 1st PC, Li-CNV gradually developed around the 1st PC spots (drawn as red circular nest). Right figure: Two-times PC group mice receive 3 intense PC shots followed by 6 additional weak PC shots (2 nd PC, drawn as white circles) around existing Li-CNV on the 7th day of 1st PC. The laser conditions for 1st and 2 nd PC are shown below the two figures.

In this study, we separated Li-CNV models into two groups using different laser conditions, as shown in Fig. 2. Briefly, the first group received 3 intense PC shots (1st PC) altogether (**one-time PC group**), while the other group received 3 intense PC shots (1st PC) followed by 6 additional weak PC shots (2 nd PC) around existing Li-CNV on the 7th day after 1st PC (**two-times PC group**). We followed the method of creating Li-CNV model established and reported by our predecessor, Nakagawa et al. [11].

The 1st PC (100-μm spot size, 100-ms pulse duration, and 200-mW incident power) is a photocoagulation which causes rupture of Bruch's membrane and leads to CNV development. The 2 nd PC (200-μm spot size, 200-ms pulse duration, and 80-mW incident power) is a photocoagulation using a weak PC that does not cause rupture in the Bruch's membrane and allows the release of endogenous FGF2 by laser-damaged cells.

### 2.3 Immunohistochemistry

#### 2.3.1 Flat-mount specimens

To investigate the synergy of CNV involved cells and certain growth factors, we performed an immunohistochemical assessment. After sacrificing the mice on the indicated days, their eyes were immediately enucleated and fixed in 4 % paraformaldehyde/PBS (phosphate-buffered saline) for 30 min. The choroid/RPE complex was isolated and permeabilized with 0.5 % Triton X-100, 20 % DMSO and 0.6 % Block Ace blocking reagent (DS Pharma Biomedical, Osaka, Japan) dissolved in PBS for 3 h at room temperature and subsequently used for immunolabeling. The following antibodies were used for immunolabeling of flat-mounted choroidal preparations: i) anti-CD31 monoclonal antibody (rat anti-mouse CD31, MEC 13.3, 1:500; BD Biosciences Pharmingen, San Diego, CA, USA) to detect blood vessels with Alexa Fluor-488 secondary antibody (1:500); ii) *anti*-PDGFRα+β monoclonal antibody (rabbit recombinant human PDGFRα+PDGFRβ antibody [Y92]-C-terminal (ab32570, 1:500, Abcam, Cambridge, MA, USA) to detect pericyte with Alexa Fluor-488secondary antibody (1:500); ⅲ) *anti*-α-SMA monoclonal antibodies Cy3 conjugate(anti-mouse α-SMA clone 1A4,1:500, Sigma, St. Louis, MO, USA) to detect smooth muscle cell and RPE cell activity. The primary antibodies were incubated at 37 °C for 2 h, and the secondary antibodies were incubated for another 2 h at 37 °C. After thorough washing, the choroid/RPE complex was then flattened and mounted with DAPI Fluoromount-G® (Southern Biotech, Birmingham, AL, USA) and cover-slipped.

#### 2.3.2 Frozen sections

After sacrificing the mice on the indicated days, their eyes were enucleated and immediately frozen on dry ice with embedded in Tissue-Tek®︎ O.C.T Compound (Sakura Finetek USA, Inc., Torrance, California, USA) and stored at −80 °C. Frozen sections were cut to 8-μm thickness with a cryostat (Leica CM3050S1; Leica Biosystems, Nuβloch, Germany). The antibodies which were used for frozen sections overlapped with those of flat-mount specimens except *anti*-FGF2 monoclonal antibody (rabbit anti-human FGF2, ab92337, 1:1000, Abcam, Cambridge, MA, USA) with Alexa Fluor-488 secondary antibody (1:500). The primary antibodies were incubated at 37 °C for 1 h, and the secondary antibodies were incubated for another hour at 37 °C. After a thoroughly washing, the frozen sections were mounted with DAPI Fluoromount-G® (Southern Biotech, Birmingham, AL, USA) and cover-slipped.

The flat-mounts specimens and the frozen sections were observed using a confocal scanning microscope (FV3000; EVIDENT, Tokyo, Japan). Immunolabeled images were obtained with a charge-coupled device camera and captured with Basler (Ahrensburg, Germany) microscopy software.

### 2.4 Optical coherence tomography angiography imaging (OCTA imaging)

OCTA was performed using RS-3000 Advance (NIDEK Co., Aichi, Japan). A mouse-specific adapter with a power of 10 diopters was installed and a platform was set to keep the animal in a stable position in front of the RS-3000 Advance, similar to previous studies [[12], [13], [14]]. For OCTA, eight linear B-scans were acquired from 256 cross-sectional locations, and retinal areas measuring 900 × 900 μm area which were centered on the CNV lesion and scanned, focusing on the choroidal vasculature. The left eye of each mouse wore a plain hard contact lens (UNICON, Osaka, Japan) to prevent corneal drying and unwanted cataract formation. We followed methods to observe vessel changes within the same individuals using OCTA established by Nakagawa et al. [11]. Li-CNV images were acquired at 7, 14, 21, and 28 days post-PC using the follow-up function with dominant OCTA software to observe vessel changes within the same individual over time.

### 2.5 Real-time quantitative polymerase chain reaction (real-time qPCR)

After sacrificing the mice on the indicated days, the retina-choroid complex was carefully isolated from their eyes and immediately stored in RNAlater® solution (Thermo Fisher Scientific, Waltham, MA, USA) to prevent degradation. The isolated retina-choroid complex was homogenized using a Biomasher tissue grinder (Nippi Inc., Tokyo, Japan). Total RNA was isolated using the RNeasy Plus Mini® Kit (Qiagen, Venlo, The Netherlands), according to the manufacturer's protocol. Total RNA was reverse-transcribed to generate cDNA using SuperScript™ IV VILO™ Master Mix (Thermo Fisher Scientific). The reverse-transcribed cDNA was subsequently subjected to real-time qPCR using the Thermal Cycler Dice® Real Time System II (TAKARA Bio, Otsu, Shiga, Japan). All reactions were prepared in a total volume of 25 μL using the TB Green® Premix Ex Taq™II PCR Kit (TAKARA Bio, Otsu, Shiga, Japan), following the manufacturer's protocol. Gene expression levels were calculated using the 2^−ΔΔCt^ method, GAPDH was used as a reference gene, and the results were presented as relative expression to control. Untreated WT mice were used as controls. (FGF2-tg mice: n = 5 or 6, WT mice: n = 5 or 6, respectively).

The target sequences used in this study were amplified using the following primers:

**FGF2**; (forward) 5′- GTGCCAACCGGTACCTTGCTA -3′ and.

(reverse) 3′- TCAGTGCCACATACCAACTGGAG -5′

FGF Receptor1 (**FGFR1)**; (forward) 5′- AGACTGGGAGCCGTGATGTG -3′ and.

(reverse) 3′- CCCATGGGTAAATCTCTAGTAACGA -5′

**VEGF-A**; (forward) 5′- AACCCATTCCTGGCCCTGA -3′ and.

(reverse) 3′- GATCCACAAAGCATGCCATGTC -5′

PDGF Receptor Beta (**PDGFRβ)**; (forward) 5′- GAACGACCATGGCGATGAGAC -3′ and.

(reverse) 3′- GGCATCGGATAAGCCTCGAA -5′

**Ang1**; (forward) 5′- CAGCCACAAAGCCTTAGTGACTTTC -3′ and.

(reverse) 3′- ATCCTGTAATGTCGGCACATACCTC -5′

**apelin**; (forward) 5′- GAGTTGCAGCATGAATCTGAGG -3′ and.

(reverse) 3′- TCTGGAGGCAACATCAGTGG -5′

**GAPDH**; (forward) 5′- TGTGTCCGTCGTGGATCTGA-3′ and.

(reverse) 3′- TTGCTGTTGAAGTCGCAGGAG -5′

### 2.6 Quantification of CNV size

The size of Li-CNV at 3, 7, 14, 21, and 28 days post-initial and 2 nd PC on CD31^+^ Immunolabeled imaging of flat-mounted choroidal preparations which were captured by a confocal scanning microscope were measured using Fiji software [15]. Additionally, the size of the Li-CNV was measured at 7, 14, 21 and 28 days after designated PC by OCTA imaging. The follow-up function with dominant OCTA software to observe vessel changes in the same individual over time was used for this evaluation. To confirm the change in the size of Li-CNV, we examined the size ratio at 14 and 28 days post-PC by setting the size of Li-CNV at 7 days post-PC to 100 % using OCTA imaging. (FGF2-tg mice: n = 6, WT mice: n = 6, respectively).

### 2.7 Statistical evaluations

Statistical calculations were performed using JMP software (SAS Institute, Inc., Cary, NC, USA). Data are presented as means ± SD or medians (first quartile to third quartile), depending on whether the data had a normal distribution, as assessed by Bartlett's test. Statistical analyses of the size of the Li-CNV were performed using the non-parametric Wilcoxon signed-rank test. Statistical analyses of the mRNA expression levels in real-time qPCR and the regression ratio of Li-CNV size compared to Li-CNV size at day7 post-PC were performed using non-parametric Steel-Dwass multiple tests. A p values < 0.05 was considered statistically significant.

## 3 Results

### 3.1 The size of Li-CNV

#### 3.1.1 One-time PC group

**Fig. 3:**
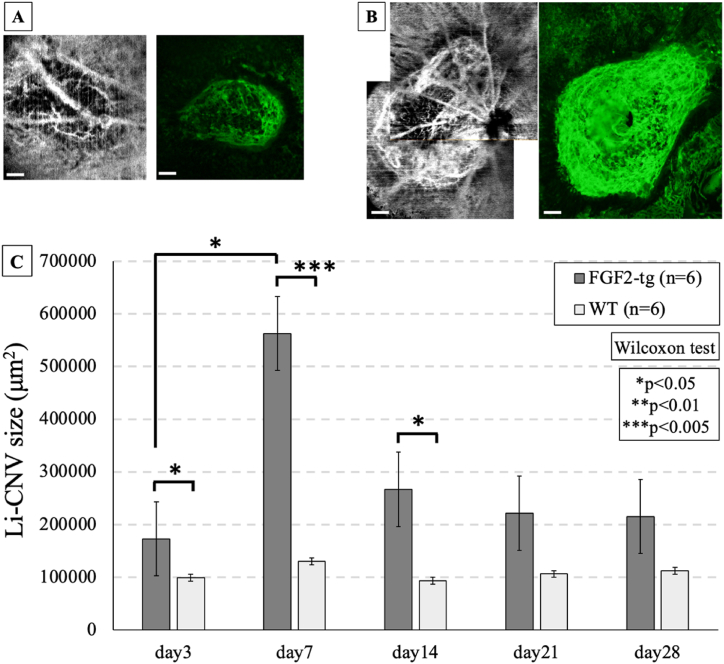
One-time PC group of FGF2-tg and WT mice at 7 days post-1st PC. **A:** Left is OCTA imaging in one-time PC group of WT mouse at 7 days post-1st PC. Li-CNV was observed as a highly signaled lesion. Right is Li-CNV of the same mouse as the left figure and was immunolabeled with anti-rat CD31 antibody (green). Scale bar = 100 μm **B:** Left is OCTA imaging in one-time PC group of FGF2-tg mouse at 7 days post-1st PC. Compared to A figures, Li-CNV shows a more clearly defined highly signaled lesion and seems larger in size. Right is Li-CNV of the same mouse as shown in the left figure and immunolabeled with anti-rat CD31 antibody (green). Scale bar = 100 μm **C:** This figure represents the time-course change in Li-CNV size in FGF2-tg and WT mice. At day 3, 7, and 14 post-1st PC, the size of Li-CNV in FGF2-tg mice were significantly larger than that in WT mice on the same days. In contrast, there were no differences in the size of Li-CNV at day 21 and 28 post-1st PC between FGF2-tg and WT mice. Additionally, the size of Li-CNV in FGF2-tg mice at day 7 post-1st PC was significantly larger than that in FGF2-tg mice at day 3 post-1st PC. (FGF2-tg mice: n = 6, WT mice: n = 6, respectively).

We measured the time-course changes in the size of Li-CNV by CD31^+^ immunolabeled imaging of flat-mounted choroidal preparations. As a results, we found that the Li-CNV size reached its maximum at 7 days post-1st PC and began to decrease thereafter, as show in Fig. 3C. Moreover, Li-CNV size in FGF2-tg mice was significantly larger than that in WT mice at 3, 7, and 14 days post-1st PC (p = 0.0153∗, p = 0.0039∗, and p = 0.0309∗, respectively). In contrast, there were no differences in Li-CNV size between FGF2-tg and WT mice at day 21 and 28 post-1st PC (p > 0.05).

#### 3.1.2 Two-times PC group

**Fig. 4:**
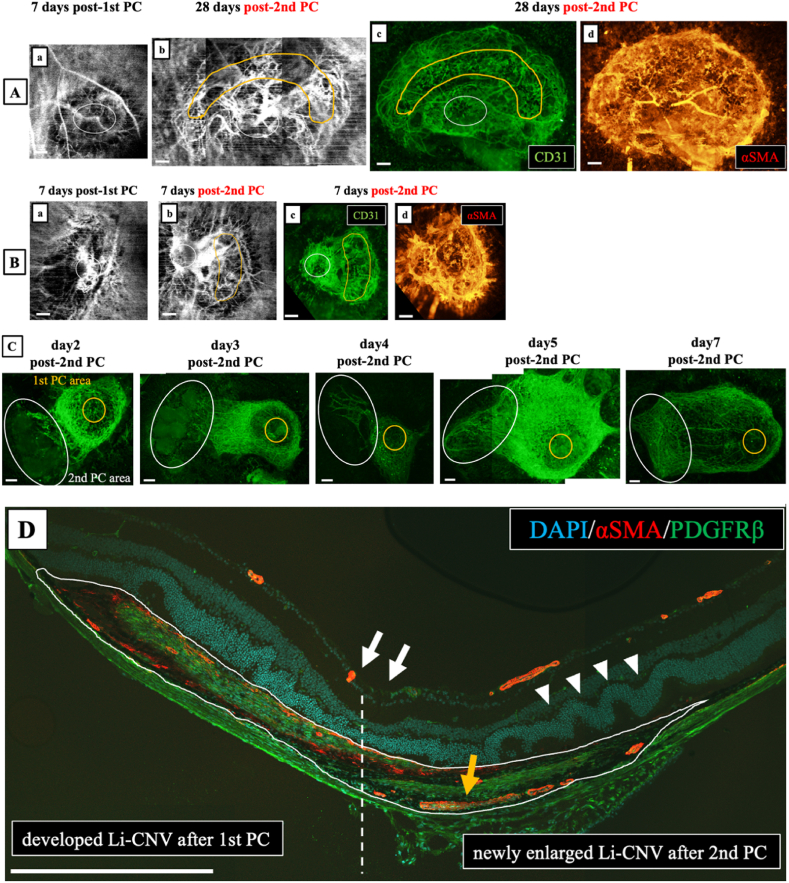
Immunolabeled imaging. **A) a)** OCTA imaging is Li-CNV in two-times PC group of FGF2-tg mouse at 7 days post-1st PC. The white circle indicated to 1st PC area. Scale bar = 100 μm **b, c)** Comparison of OCTA and immunolabeled imaging using anti-rat CD31 antibody (green) from same mouse at 28 days post-2nd PC. Li-CNV enlarged toward to 2 nd PC area (yellow circle). Scale bar = 100 μm **d)**A flat-mounted choroid preparation from of the same mouse as shown in the figure Bb) and Bc) immunolabeled with anti-mouse α-SMA antibody (red). New vessels branching toward to 2 nd PC area surrounded α-SMA positive smooth muscle cells were observed. A α-SMA detected smooth muscle cells and RPE cell activity. Scale bar = 100 μm. B**) a)** OCTA imaging is Li-CNV in two-times PC group of WT mouse at 7 days post-1st PC. The white circle indicated to 1st PC area. Scale bar = 100 μm **b, c)** Comparison of OCTA and immunolabeled imaging using anti-rat CD31 antibody (green) from same mouse at 7 days post-2nd PC. Li-CNV enlarged toward to 2 nd PC area (yellow circle). Scale bar = 100 μm **d)** A flat-mounted choroid preparation from of the same mouse as shown in the figure Cb) and *Cc*) immunolabeled with anti-mouse α-SMA antibody (red). In contrast to figure Bd), no vessels surrounded α-SMA positive smooth muscle cells were observed. A α-SMA detected smooth muscle cells and RPE cell activity. Scale bar = 100 μm. C) These figures illustrate the time-course changes in Li-CNV in two-times PC group of FGF2-tg mice, as observed through immunolabeled imaging of flat-mounted choroidal preparations using from different FGF2-tg mice. Around 3 days post-2nd PC, new vascular networks developed from the existing Li-CNV in the 1st PC area (yellow circle) instead of the 2 nd PC area (white circle), and then, neovascular vessels extended toward the 2 nd PC area by 7 days post-2nd PC. Scale bar = 100 μm. D) A frozen section from two-times PC group of FGF2-tg mouse at 7 days post-2nd PC was immunolabeled with anti-rabbit PDGFRβ antibody (green), anti-mouse α-SMA (red) antibody, and DAPI (blue). White arrows indicate 1st PC spots. White arrowheads indicate 2 nd PC spots. Li-CNV separated two areas, one is developed Li-CNV after 1st PC (left side from dotted line) and another is newly enlarged Li-CNV after 2 nd PC (right side from dotted line). Vessels surrounded by PDGFRβ positive pericytes and α-SMA positive smooth muscle cells (yellow arrows) were observed in newly enlarged Li-CNV after 2 nd PC. Scale bar = 500 μm.

The size of Li-CNV in the two-times PC group was significantly larger than that in the one-time PC group in both FGF2-tg and WT mice, as shown in Fig. 4A and B. By 7 days post-2nd PC, angiogenesis occurred from the existing Li-CNV in the 1st PC area instead of the 2 nd PC area and then, neovascular vessels branched and extended toward the 2 nd PC area in both FGF2-tg and WT mice, as shown in Fig. 4C. The two-times PC group showed enlargement of the neovascularization area and augmented mature vessels surrounded by PDGFRβ positive pericytes and α-SMA positive smooth muscle cells, as shown in Fig. 4D. On the other hand, in cases where only 6 weak PC shots (2 nd PC) were performed in both FGF2-tg and WT mice, no neovascularization occurred.

### 3.2 Imaging of Li-CNV in FGF2-tg and WT mice using OCTA

#### 3.2.1 One-time PC group

**Fig. 5:**
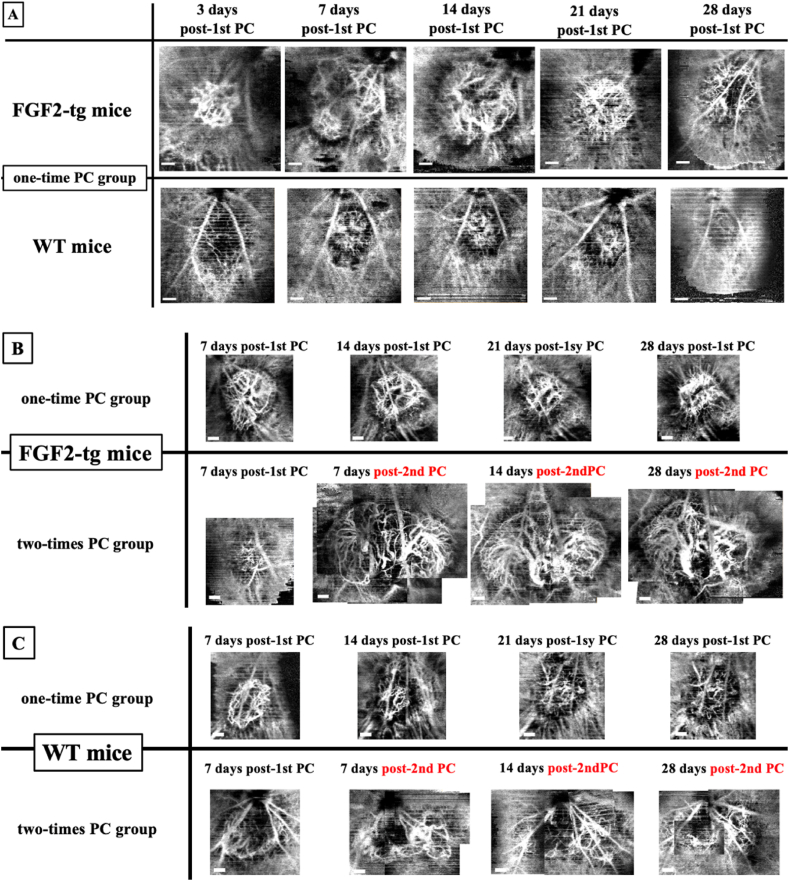
The time-course changes of Li-CNV in FGF2-tg and WT mice observed by OCTA. We observed Li-CNV in FGF2-tg and WT mice over time in the same individual until 28 days post-1st PC in the one-time PC group and post-2nd PC in the two-times PC group using OCTA.**A.** In both the one-time PC group of FGF2-tg and WT mice, Li-CNV size reached its maximum at 7 days post-1st PC and highly signaled lesion of Li-CNV gradually became indistinct by 28 days post-1st PC. Scale bar = 100 μm **B.** The one-time PC group of FGF2-tg mice clearly showed a decrease in Li-CNV size by 28 days post-1st PC compared with those of the Li-CNV size at 7 days post-1st PC. In contrast, in two-times PC group of FGF2-tg, highly signaled lesion of Li-CNV was illustrated clearly and Li-CNV size maintained until 28 days post-2nd PC. Scale bar = 100 μm, **C.** The highly signaled lesion of Li-CNV gradually became indistinct by 28 days post-1st PC in the one-time PC group of WT mice. In the two-times PC group of WT mice, Li-CNV enlarged at 7 days post-2nd PC compared with those of the Li-CNV at 7 days post-1st PC and CNV networks of both groups gradually became indistinct by 28 days post-2nd PC. Scale bar = 100 μm.

Using OCTA, as shown in Fig. 5A, we observed Li-CNV of FGF2-tg and WT mice over time in the same individual until 28 days post-1st PC. In both one-time PC group of FGF2-tg and WT mice, Li-CNV regression started at 7 days post-1st PC as far as CNV size were concerned, and then Li-CNV was not observed clearly at 28 days post-1st PC.

#### 3.2.2 Two-times PC group

We observed Li-CNV in both the one-time PC and two-times PC group in FGF2-tg over time in the same individual until 28 days post-PC, as shown in Fig. 5B. In the two-times PC group of FGF2-tg mice, Li-CNV size was maintained until 28 days post-2 (a total of 35 days from 1st PC), and neither expanded nor regressed from 7 days. In contrast, in the two-times PC group of WT mice, Li-CNV enlarged at 7 days post-2nd PC compared with those of the Li-CNV at 7 days post-1st PC and CNV networks of both groups gradually became indistinct by 28 days post-2nd PC, as shown in Fig. 5C.

### 3.3 Real-time qPCR

#### 3.3.1 One-time PC group

**Fig. 6:**
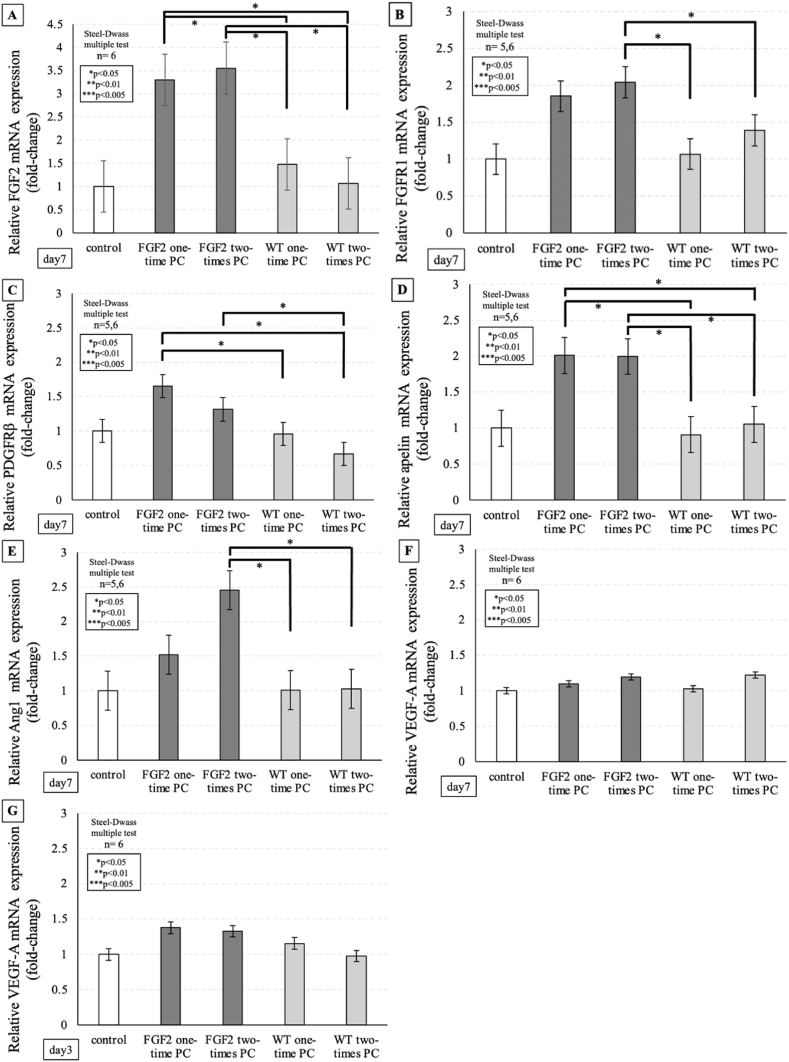
The real-time qPCR in FGF2-tg and WT mice. Gene expression levels were calculated using the 2^−ΔΔCt^ method, GAPDH was used as a reference gene, and the results were presented as relative expression to the control. Untreated WT mice were used as controls. **A:** FGF2 mRNA expression level in the one-time PC group of FGF2-tg mice at 7 days post-1st PC was significantly higher than that in WT mice on the same day. Additionally, FGF2 mRNA expression level in the two-times PC group of FGF2-tg mice at 7 days post-2nd PC was significantly higher than that in WT mice on the same day. On the other hand, there was no significant increase in FGF2 mRNA expression level at 7 days post-2nd PC in WT mice compared with the untreated WT control mice on the same day. (FGF2-tg mice: n = 6, WT mice: n = 6, respectively), **B:** FGFR1 mRNA expression level in the two-times PC group of FGF2-tg mice at 7 days post-2nd PC was significantly higher than that in WT mice on the same day. On the other hand, there was no significant increase in FGFR1 mRNA expression level at 7 days post-2nd PC in WT mice compared with the untreated WT control mice on the same day. (FGF2-tg mice: n = 5 or 6, WT mice: n = 5 or 6, respectively), **C:** PDGFRβ mRNA expression level in the one-time PC group of FGF2-tg mice at 7 days post-1st was significantly higher than that in WT mice on the same day. Additionally, PDGFRβ mRNA expression level in the two-times PC group of FGF2-tg mice at 7 days post-2nd PC was significantly higher than that in WT mice on the same day. On the other hand, there was no significant increase in PDGFRβ mRNA expression level at 7 days post-2nd PC in WT mice compared with the untreated WT control mice on the same day. (FGF2-tg mice: n = 5 or 6, WT mice: n = 5 or 6, respectively), **D:** Apelin mRNA expression level in the one-time PC group of FGF2-tg mice at 7 days post-1st was significantly higher than that in WT mice on the same day. Additionally, apelin mRNA expression level in the two-times PC group of FGF2-tg mice at 7 days post-2nd PC was significantly higher than that in WT mice on the same day. On the other hand, there was no significant increase in apelin mRNA expression level at 7 days post-2nd PC in WT mice compared with the untreated WT control mice on the same day. (FGF2-tg mice: n = 5 or 6, WT mice: n = 5 or 6, respectively), **E:** Ang1 mRNA expression level in two-times PC group of FGF2-tg mice at 7 days post-2nd PC was significantly higher than those in WT mice on the same day. On the other hand, there was no significant increase in Ang1 mRNA expression level at 7 days post-2nd PC in WT mice compared with the untreated WT control mice on the same day. (FGF2-tg mice: n = 5 or 6, WT mice: n = 5 or 6, respectively), **F:** There was no significant increase in VEGF-A mRNA expression level at 7 days post-1st PC and at 7 days post-2nd PC in both of FGF2-tg and WT mice compared with the untreated WT control mice on the same day. (FGF2-tg mice: n = 6, WT mice: n = 6, respectively), **G.** There was no significant increase in VEGF-A mRNA expression level at 3 days post-1st PC and at 3 days post-2nd PC in both of FGF2-tg and WT mice compared with the untreated WT control mice on the same day. (FGF2-tg mice: n = 6, WT mice: n = 6, respectively).

Real-time qPCR, as shown in Fig. 6A–G, confirmed that FGF2, PDGFRβ, and apelin mRNA expression levels in FGF2-tg mice were significantly higher than in WT mice at 7 days post-1st PC (p = 0.0257∗ p = 0.0398∗, and p = 0.0405∗, respectively). On the other hand, there was no significant increase in VEGF-A mRNA expression levels at 3 and 7 days post-1st PC between FGF2-tg and WT mice (p > 0.05).

#### 3.3.2 Two-times PC group

Real-time qPCR confirmed, as shown in Fig. 6A–G, that FGF2, FGFR1, Ang1, and apelin mRNA expression levels were significantly higher in FGF2-tg at 7 days post-2nd PC than in WT mice at 7 days post-1st PC (p = 0.0257∗, p = 0.0257∗, p = 0.0405∗, and p = 0.0405∗, respectively). Additionally, FGF2, FGFR1, PDGFRβ, apelin, and Ang1 mRNA expression levels in FGF2-tg at 7 days post-2nd PC were significantly higher in FGF2-tg at 7 days post-2nd PC than in WT mice at 7 days post-2nd PC (p = 0.0261∗, p = 0.0494∗, p = 0.0405∗, p = 0.0261∗, and p = 0.0261∗ respectively). On the other hand, there was no significant increase in FGF2, FGFR1, PDGFRβ, Ang1, and apelin mRNA expression levels at 7 days post-2nd PC in WT mice (p > 0.05).

### 3.4 Size ratio of Li-CNV compared to Li-CNV size at day 7 post-PC

**Fig. 7:**
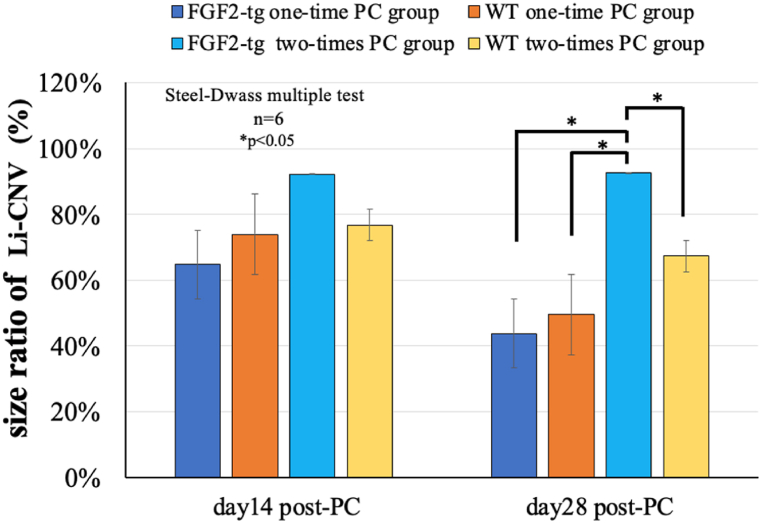
The size ratio of Li-CNV compared to Li-CNV size at day7 post PC. To confirm the change in Li-CNV size, we examined the size ratio at 14 and 28 days post-PC by setting the Li-CNV size at 7 days post-PC to 100 % using OCTA imaging. There was no difference in the size ratio of Li-CNV at day 14 post-PC in any of the groups. On the other hand, the size ratio of Li-CNV at day 28 post-2nd PC in two-times PC group of FGF2-tg mice was statistically higher than that in the one-time PC group of FGF2-tg mice. Furthermore, the size ratio of Li-CNV at day 28 post-2nd PC in the two-times PC group of FGF2-tg mice was statistically higher than that in WT mice. In other words, the Li-CNV size in two-times PC group of FGF2-tg mice was maintained until 28 days post-2nd PC. (FGF2-tg mice: n = 6, WT mice: n = 6, respectively).

To confirm the change in Li-CNV size, we examined the size ratio at 14 and 28 days post-PC by setting the Li-CNV size at 7 days post-PC to 100 % using OCTA imaging, as shown in Fig. 7. There was no difference in the size ratio of Li-CNVs at day 14 post-PC in any of the groups. On the other hand, the size ratio of Li-CNV at day 28 post-2nd PC in two-times PC group of FGF2-tg mice was statistically higher than that in the one-time PC group of FGF2-tg mice. Furthermore, the size ratio of Li-CNV at day 28 post-2nd PC in the two-times PC group of FGF2-tg mice was statistically higher than that in WT mice. In other words, the Li-CNV size in two-times PC group of FGF2-tg mice was maintained until 28 days post-2nd PC.

## 4 Discussion

The laser-induced choroidal neovascularization (Li-CNV) model, which has proven to be indispensable for studying exudative age-related macular degeneration (AMD). According to the literatures for Li-CNV [11], CNV comprises of three phases. These phases include the initial inflammatory response, which marks the initiation of the growth phase (**1st phase**); second, the development of CNV, where neovascular vessels are observed around 3 days post-PC and then CNV size reaches a maximum around 7 days post-PC (**2nd phase**); and lastly, CNV regresses and reduces in size (**3rd phase**). Although previous research has indicated that FGF2 plays a crucial role in neovascularization and vessel maturation and stabilization, suggesting its influence on all three phases [4,[16], [17], [18]], the exact relationship between FGF2 and CNV remains unclear. One of the barriers to research in this field is the lack of investigation for time-course changes using the same individual. Fortunately, we have OCTA, a non-invasive and useful tool compared to conventional angiographies, which allows us to visualize the retinal and choroidal vasculature without invasive dye injection or sacrificing animals. This device allows researchers to observe vessel changes in the same individual over time, which is a significant breakthrough in this field of research. Moreover, we have FGF2-tg mice which are transgenic mice with a rhodopsin promoter/FGF2 gene fusion that expresses high levels of FGF2 only in the retinal photoreceptors (Fig. 1A). Originally, naïve FGF2-tg mice did not show any pathological retinal neovascularization nor other retinal structural abnormalities including vasculatures (Fig. 1B) [10]. Interestingly, in FGF2-tg, FGF2 significantly affects angiogenesis only when FGF2 is released into the extracellular space by injuring its photoreceptor layer via laser photocoagulation. In fact, when FGF2-tg mice received laser-induced rupture of Bruch's membrane, they developed larger choroidal neovascularization than their littermate controls (Fig. 3A–C), similar to a previous study [9]. In our study, we evaluated the effects of FGF2 on angiogenesis in mice using the OCTA and FGF2-tg mice. Specifically, we examined vessel changes over time and compared these changes between FGF2-tg and WT mice. Our goal is to gain better understandings of the dynamic role that FGF2 plays in the three phases of CNV as described above.

Previous studies have reported that FGF2 is a powerful angiogenic stimulators [[16], [17], [18]]. Hu et al. [19] indicated that FGF2 mRNA expression significantly increased after PC in WT mice and substantial increases in FGF2 mRNA expression were observed in the RPE/choroidal samples as an initial inflammatory response which is thought as first phase of CNV. Moreover, Hu et al. [18] also reported a secondary increase in FGF2 mRNA expression was detected at 1 week post-PC (2nd phase of CNV) in the RPE/choroidal samples. Onimaru et al. [20] reported that an initial increase in FGF2 stimulated angiogenesis by activating VEGF-A expression in endothelial and stromal cells. On the other hand, Onimaru et al. [20] also reported that a secondary increase in FGF2 promotes vascular maturation by upregulating PDGFRβ expression in mural cells (MCs). Therefore, FGF2 could potentially play a role in angiogenesis during 1st to 2nd phase of CNV process, and in vascular maturation during 2nd to 3rd phase of CNV process. To prove this hypothesis, for 1st to 2nd phase of CNV process, we developed a Li-CNV model using one-time intense PC to observe the time-course change of vessel and investigate the factors involved in angiogenesis using real-time qPCR. Additionally, for 2nd to 3rd phase of CNV process, we developed a Li-CNV model by adding endogenous supply of FGF2 using weak PC around existing Li-CNV at 7 days after initial intense PC to observe the change of vessel thereafter and investigate the factors involved in vessel maturation using real-time qPCR.

### 4.1 Role of FGF2 in 1st to 2nd phase of CNV process

To investigate the role of FGF2 in 1st to 2nd phase of CNV process, we developed a Li-CNV model using one-time intense PC and observed that the size of Li-CNV at 7 days post-1st PC in FGF2-tg mice was significantly larger than that in WT mice (p = 0.0039∗, Fig. 3C). This result was compatible to those in Yamada et al. [9,10]. As a phenomenon, FGF2 augmented CNV development. As a results of real-time qPCR, FGF2 mRNA expression in FGF2-tg mice was significantly higher than that in WT mice at 7 days post-1st PC (Fig. 6A). After PC in WT mice, we observed an increase in VEGF-A mRNA expression, leading us to anticipate higher VEGF-A mRNA expression levels in FGF2-tg mice lead by FGF2 mRNA expression. However, contrary to previous reports and our expectations, there was no significant increase in VEGF-A mRNA expression at 3 and 7 days post-PC in both FGF2-tg and WT mice (Fig. 6F and G). Based on these findings, we hypothesize that the development of new blood vessels in FGF2-tg mice is promoted by VEGF-A-independent FGF2 signaling pathway. To clarify this hypothesis, we observed apelin mRNA expression. Apelin was initially identified as a ligand for the orphan G-protein-coupled receptor named APJ, which is highly expressed in endothelial cells at the tips of growing capillaries, where APJ mediates the paracrine effects of apelin on endothelial cells [[21], [22], [23], [24]]. It has been previously reported that apelin stimulates the proliferation and migration of retinal endothelial cells, resulting in enhanced vascular tube formation [25]. Furthermore, apelin expression is upregulated by FGF2, indicating crosstalk with major angiogenic factors. Interestingly, the intracellular signaling pathways of apelin are involved in exerting cell proliferative effects independently of the PKC/Raf/MEK pathway of VEGF through the activation of Akt/mTOR/p70S6 kinase. In our study, a substantial increase in apelin mRNA expression was found at 7 days post-1st PC in the one-time PC group of FGF2-tg compared to that in WT mice (p = 0.0405∗, Fig. 6D). Based on our results, we considered that excessive ECs proliferation of Li-CNV in FGF2-tg mice may be promoted by the apelin/APJ system that is independent of VEGF signaling pathway.

### 4.2 Role of FGF2 in 2nd to 3rd phase of CNV process

We observed the time-course change in Li-CNV in FGF2-tg and WT mice over time in the same individual until 28 days post-1st PC using OCTA. In both the one-time PC group in FGF2-tg and WT mice, Li-CNV regression started at 7 days post-1st PC as far as CNV size was concerned. Although the size of Li-CNV at 7 days post-1st PC in FGF2-tg mice was significantly larger than that in WT mice, it seems curious that there was no difference in the timing of the vessel transition from 2nd phase to 3rd phase of CNV process (Fig. 7). We formally thought that the timing of the vessel's transition from 2nd phase to 3rd phase would be delayed if the effects of FGF2, which promotes vessel development, persisted; however, the outcomes have been indicated otherwise. One possible reason for this is that the effect of excess FGF2, which is localized around new vessels, no longer exists due to its consumption or degradation. To determine the mechanism how FGF2 acts against already existing CNV, we performed additional experiment. This experiment was conducted to determine how existing neovascular vessels change when additional FGF2 supplied by a weak PC. The additional PC applied as 2 nd PC is a photocoagulation using a weak PC that does not cause a rupture in Bruch's membrane and allows the release of endogenous FGF2 by laser-damaged cells. By 7 days post-2nd PC, angiogenesis occurred from the existing Li-CNV in the 1st PC area instead of the 2 nd PC area, and then, neovascular vessels branched and extended toward the 2 nd PC area in both FGF2-tg and WT mice (Fig. 4A). Interestingly, in the two-times PC group of FGF2-tg mice, Li-CNV size was maintained until 28 days post-2nd PC (total of 35 days from 1st PC), and neither expanded nor regressed from 7 days (Fig. 5B). In other words, the existing neovascular network would normally have regressed, seemed to have been maintained until 28 days.

Neovascular maturation is a crucial aspect of angiogenesis and FGF2 plays a vital role in this process. Although both FGF2 and VEGF-A are powerful angiogenic stimulators, the capillaries developed by FGF2 are tightly sealed and have a distinct morphology compared to VEGF-induced capillaries [6]. Previous reports have suggested that FGF2 signaling enhances PDGF, which is centrally involved in vascular network maturation and remodeling by recruiting MCs (pericytes or smooth muscle cells) to the endothelium [26]. Recruitment and investment of MCs in neovascular vessels are crucial for vessel stabilization and maturation [27]. In the absence of FGF2, ECs became less responsive to PDGF-BB because of decreased PDGFRβ expression. In addition, ablation of PDGF-BB or PDGFRβ in mice causes MC deficiency, leading to widespread vascular leakage and perinatal mortality. While FGF2 alone can induce moderate vessel maturation, studies have shown that a combination of FGF2 and PDGF-BB synergistically promotes the formation of durable vessels that remain stable for an extended period, even after the depletion of these growth factors [28,29]. In our study, real-time qPCR results revealed that PDGFRβ mRNA expression level was significantly higher in FGF2-tg mice at 7 days post-2nd PC than in WT mice (p = 0.0405∗, Fig. 6C). Although CNV expansion stimulated by neovascularization was not observed after 7 days post-2nd PC, the existing neovascular network seemed stabilized in the two-times PC group of FGF2-tg mice. This phenomenon was confirmed by immunoblotting for α-SMA and PDGFRβ. Namely, existing neovascular vessels extending from 1st PC area to 2 nd PC area are mature vessels surrounded by PDGFRβ positive pericytes and α-SMA positive smooth muscle cells (Fig. 4D). Our results possibly suggested that when FGF2 was reintroduced at the timing of vessel transition from 2nd phase to 3rd phase of CNV process, it collaborated with PDGF-BB to enhance the maturation of pre-existing Li-CNV through remodeling by recruiting MCs, including PDGFRβ positive pericytes and α-SMA positive smooth muscle cells, rather than vessel regression. Therefore, proper timing of the additional supply of FGF2 causes not only neovascularization but also vessel maturation.

Surprisingly, in this study, the mRNA expression level of angiopoietin 1 (Ang1) in FGF2-tg mice at 7 days post-2nd PC was especially higher than those in WT mice at 7 days post-1st PC and at 7 days post-2nd PC (Fig. 6E). There was no significant increase in Ang1 mRNA expression level at 7 days post-1st PC or at 7 days post-2nd PC in WT mice. Inducing the formation of blood vessels and maintaining their integrity are two separate processes that are equally crucial for establishing a stable vascular system. As we previously mentioned, during the process of blood vessel formation, ECs recruit supportive MCs by releasing PDGF-BB. Another process, maintaining blood vessel integrity, involves MCs attaching to ECs, resulting in a structurally sound blood vessel. It is believed that cell adhesion between MCs and ECs is triggered when Ang1, which is produced by MCs, stimulates Tie2 which is a receptor tyrosine kinase on ECs. Endothelial FGF2 signaling is crucial for the maintenance of blood vessels by reinforcing VE-cadherin at endothelial cell-cell junctions, which is attained by stimulating Ang1. When FGF2 signaling impedes the VE-cadherin complex, which disassembles intercellular junctional adhesion, it causes loss of ECs and severe reduction in endothelial barrier function, ultimately leading to the disintegration of vasculature [30]. These findings suggest that the maintaining blood vessels requires ongoing FGF2 stimulation. On the other hand, VEGF inhibits VE-cadherin adhesion through the VEGF type 2 receptor (VEGFR2) and increases vascular permeability, while Ang1 counteracts this VEGF-induced inhibition of VE-cadherin adhesion [31]. Therefore, our results possibly suggested that the reintroduction of FGF2 at the timing of vessel transition from 2nd to 3rd phase of CNV process initially collaborated with PDGFRβ to facilitate the maturation of pre-existing blood vessels. Furthermore, it may work in conjunction with Ang1 to promote cell adhesion and maintain vessel structural integrity, and finally achieve vessel stabilization.

Although *anti*-VEGF therapy has emerged as a standard treatment for neovascular age-related macular degeneration (AMD), it is well known that there are several patients whose CNV are not effective for this drug. Additionally, the long-term use of *anti*-VEGF therapies sometimes causes impaired treatment due to acquired drug resistance [32,33], thus it is eagerly required to search for new drugs for AMD other than *anti*-VEGF drugs. The apelin-APJ system is independent of VEGF signaling and may be a candidate for a new therapeutic target for AMD. Moreover, *anti*-VEGF therapy may result in the temporary regression of neovascular vessels in AMD, it also triggers tissue hypoxia and neovascular vessel regeneration, causing gradual tissue damage due to CNV's fragile intercellular adhesion, high permeability, and leakage of pro-inflammatory factors into the surrounding tissues. Eventually, this cycle results in retinal fibrosis and irreversible loss of vision [34]. The concept of “vascular normalization” has gained significant attention for breaking this cycle [35]. Vascular normalization involves the maturation of neovascular vessels to stabilize them, thereby providing essential oxygen to tissues and improving vascular permeability, which in turn reduces excessive inflammatory responses in surrounding tissues [36]. This concept has been studied in clinical trials in various fields [[35], [36], [37]]. As shown in the present study, our data suggest a new approach for developing functional non-leaky blood vessels by regulating the permeability of newly formed vessels by FGF2. The timing of FGF2 administration is crucial for achieving this concept while minimizing its angiogenic effects. In research for the timing when to induce FGF2 at the right time should be studied in the future. We also suggest that combining therapies that target both VEGF-dependent and VEGF-independent pathways may produce a synergistic effect. This approach can potentially enhance the overall efficacy of CNV treatment and decrease the risk of disease recurrence.

It is important to acknowledge the limitations of our study. First, the limited sample size prevented us from fully assessing the reliability and accuracy of our results. Further research is necessary to confirm our results in larger and more diverse populations, and to fully explore the potential of FGF2 pathways in clinical settings. Second, we did not use transmission electron microscopy (TEM) to visualize vessel tube formation, which makes it difficult to confirm the presence of pericytes, smooth muscle cells, or cell adhesion. In spite of not using TEM in our study, the finding that existing Li-CNV extending from 1st PC area to 2nd PC area was stained with PDGFRβ and α-SMA by immunolabeling must support our hypothesis that FGF2 not only works for strong proangiogenic effects but also for maintenance and maturation of existing blood vessels. Third, we evaluated the mRNA expression levels using real-time qPCR, but there is a possibility of lacking to check FGF2 signal activation by phosphorylation. It is because in FGF2-tg mice have unique form storing FGF2 in the photoreceptor cells and FGF signaling cascades are not clear. In return, we propose that monitoring changes in apelin mRNA expression levels using real-time qPCR, which is downstream of FGF2 signaling, may support our hypothesis. To enhance our understanding of angiogenesis and its underlying factors, future studies should consider these limitations and aim to replicate the clinical scenario more accurately while investigating interactions among various vessel growth factors. By addressing these limitations, we can gain a better understanding of the complex mechanisms involved in vessel maturation and stabilization which we can potentially identify new targets for therapeutic interventions in AMD.

In summary, FGF2 significantly influenced vascular ECs by contributing to vessel maturation and stabilization through the stimulation of PDGFRβ, Ang1, and apelin. Our results suggest that the roles of FGF2 on blood vessels may vary depending on the stage of angiogenesis, and it could be possible to control the regression process by affecting FGF2 in neovascular vessels at the appropriate timing.

## 5 Conclusions

FGF2 not only promotes neovascularization but also helps to maintain and stabilize pre-existing neovascular vessels. The effect of FGF2 on blood vessels can be deliberately altered based on the stage of angiogenesis. Our results indicate that if we apply FGF2 to the existing CNV by proper timing, we may promote vessel maturation and reduce vascular leakage.

## CRediT authorship contribution statement

**Yuki Hattori:** Writing – review & editing, Writing – original draft, Visualization, Validation, Supervision, Software, Resources, Project administration, Methodology, Investigation, Formal analysis, Data curation, Conceptualization. **Haruhiko Yamada:** Validation, Supervision, Project administration. **Hidetsugu Mori:** Validation, Supervision. **Shinpei Oba:** Methodology, Investigation. **Kaito Yokota:** Methodology, Investigation. **Masatoshi Omi:** Methodology, Investigation. **Yuichi Yamamoto:** Investigation. **Keiko Toyama:** Visualization, Validation, Methodology, Formal analysis. **Masayuki Ohnaka:** Supervision. **Kanji Takahashi:** Supervision. **Hisanori Imai:** Validation, Supervision.

## Declaration of competing interest

The authors declare that they have no known competing financial interests or personal relationships that could have appeared to influence the work reported in this paper.

## References

[1] Rey Sergio, Semenza Gregg L. (2010). Hypoxia-inducible factor-1-dependent mechanisms of vascularization and vascular remodelling. Cardiovasc. Res..

[2] Carmeliet P., Jain R.K. (2011). Molecular mechanisms and clinical applications of angiogenesis. Nature.

[3] Seghezzi G., Patel S., Ren C.J. (1998). Fibroblast growth factor-2 (FGF-2) induces vascular endothelial growth factor (VEGF) expression in the endothelial cells of forming capillaries: an autocrine mechanism contributing to angiogenesis. J. Cell Biol..

[4] (2012). Masahiro Murakami and Takashi Sakurai. Role of fibroblast growth factor signaling in vascular formation and maintenance: orchestrating signaling networks as an integrated system. WIREs Syst Biol Med.

[5] Senger R., Galli S.J., Dvorak A.M., Perruzzi C.A., Susan Harvey V., Dvorak H.F. (1983). Tumor cells secrete a vascular permeability factor that promotes accumulation of ascites fluid. Science.

[6] Cao R., Eriksson A., Kubo H., Alitalo K., Cao Y., Thyberg J. (2004). Comparative evaluation of FGF-2-, VEGF-A-, and VEGF-C-induced angiogenesis lymphangiogenesis, vascular fenestrations, and permeability. Circ. Res..

[7] Jih Y.J., Lien W.H., Tsai W.C., Yang G.W., Li C., Wu L.W. (2001). Distinct regulation of genes by bFGF and VEGF-A in endothelial cells. Angiogenesis.

[8] Steringer J.P., Bleicken Stephanie, Andreas Helena (2012). Phosphatidylinositol 4,5-bisphosphate (PI(4,5)P2)-dependent oligomerization of fibroblast growth factor 2 (FGF2) triggers the formation of a li- pidic membrane pore implicated in unconventional secretion. J. Biol. Chem..

[9] Haruhiko Y., Eri Y., Kwak Nohoon (2000). Cell injury unmasks a latent proangiogenic phenotype in mice with increased expression of FGF2 in the retina. J. Cell. Physiol..

[10] Ozaki H., Okamoto N., Ortega S. (1998). Basic fibroblast growth factor is neither necessary nor sufficient for the development of retinal neovascularization. Am. J. Pathol..

[11] Kazuki N., Haruhiko Y., Hidetsugu M., Keiko T., Kanji T. (2018). Comparison between optical coherence tomography angiography and immunolabeling for evaluation of laser-induced choroidal neovascularization. PLoS One.

[12] Park J.R., Choi W., Hong H.K. (2016). Imaging laser-induced choroidal neovascularization in the rodent retina using optical coherence tomography angiography. Invest. Ophthalmol. Vis. Sci..

[13] Shah R.S., Soetikno B.T., Yi J. (2016). Visible-light optical coherence tomography angiography for monitoring laser-induced choroidal neovascularization in mice detecting CNV via vis-OCTA. Invest. Ophthalmol. Vis. Sci..

[14] Maged A., Andre R., Anja H. (2016). OCT angiography in the mouse: a novel evaluation method for vascular pathologies of the mouse retina. Exp. Eye Res..

[15] Schindelin J., Carreras Arganda, Frise Erwin (2012). Fiji: an open-source platform for biological-image analysis. Nat. Methods.

[16] Cox C.M., Poole T.J. (2000). Angioblast differentiation is influenced by the local environment: FGF-2 induces angioblasts and patterns vessel formation in the quail embryo. Dev. Dynam..

[17] Deindl Elisabeth, Hoefer Imo E., Fernandez Borja (2003). Involvement of the fibroblast growth factor system in adaptive and chemokine- induced arteriogenesis. Circ. Res..

[18] Scholz D., Ziegelhoeffer T., Helisch A. (2002). Contribution of arteriogenesis and angiogenesis to postocclusive hindlimb perfusion in mice. J. Mol. Cell. Cardiol..

[19] Hu Wenzheng, Criswell Mark H., Fong Shao-Ling (2009). Differences in the temporal expression of regulatory growth factors during choroidal neovascular development. Exp. Eye Res..

[20] Mitsuho O., Yoshikazu Y., Katsuo S. (2002). Fibroblast growth factor-2 gene transfer can stimulate hepatocyte growth factor expression irrespective of hypoxia-mediated downregulation in ischemic limbs. Circ. Res..

[21] Tatemoto K., Hosoya M., Habata Y. (1998). Isolation and characterization of a novel endogenous peptide ligand for the human APJ receptor. Biochem. Biophys. Res. Commun..

[22] Kleinz M.J., Davenport A.P. (2004). Immunocytochemical localization of the endogenous vasoactive peptide apelin to human vascular and endocardial endothelial cells. Regul. Pept..

[23] del Toro R., Prahst C., Mathivet T. (2010). Identification and functional analysis of endothelial tip cell-enriched genes. Blood.

[24] Kim J., Kang Y., Kojima Y. (2013). An endothelial apelin-FGF link mediated by miR-424 and miR-503 is disrupted in pulmonary arterial hypertension. Nat. Med..

[25] Chikako H., Atsushi K., Fumi G. (2013). Biochemistry and molecular biology. Laser-induced choroidal neovascularization in mice attenuated by deficiency in the apelin-APJ system. Invest. Ophthalmol. Vis. Sci..

[26] Do Young Park, Lee Junyeop, Kim Jaeryung (2017). Plastic roles of pericytes in the blood–retinal barrier. Nat. Commun..

[27] Richardson T.P., Peters M.C., Ennett A.B., Mooney D.J. (2001). Polymeric system for dual growth factor delivery. Nat. Biotechnol..

[28] Cao R., Brakenhielm E., Li X., Pietras K. (2002). Angiogenesis stimulated by PDGF-CC, a novel member in the PDGF family, involves activation of PDGFR-alphaalpha and -alphabeta receptors. Faseb. J..

[29] Cao R., Brakenhielm E., Pawliuk R. (2003). Angiogenic synergism, vascular stability and improvement of hind- limb ischemia by a combination of PDGF-BB and FGF-2. Nat. Med..

[30] Murakami M., Nguyen L.T., Zhuang Z.W. (2008). The FGF system has a key role in regulating vascular integrity. J. Clin. Invest..

[31] Falcon B.L., Hiroya H., Koumoutsakos Petros (2009). Contrasting actions of selective inhibitors of angiopoietin-1 and angiopoietin-2 on the normalization of tumor blood vessels. Am. J. Pathol..

[32] Martin D.F., Maguire M.G., Ying G.S., CATT Research Group (2011). Ranibizumab and bevacizumab for neovascular age-related macular degeneration. N. Engl. J. Med..

[33] Martin D.F., Maguire M.G., Fine S.L. (2012). Ranibizumab and bevacizumab for treatment of neovascular age-related macular degeneration: two-year results. Ophthalmology.

[34] Wu Di, Kanda Atsuhiro, Liu Ye (2019). Galectin-1 promotes choroidal neovascularization and subretinal fibrosis mediated via epithelial- mesenchymal transition. Faseb. J..

[35] Rakesh K. Jain (2005). Normalization of tumor vasculature: an emerging concept in antiangiogenic therapy. Science.

[36] Kidoya Hiroyasu, Naito Hisamichi, Takakura Nobuyuki (2010). Apelin induces enlarged and nonleaky blood vessels for functional recovery from ischemia. Blood.

[37] Kayoko H., Yang Yunlong, Takahiro S. (2020). Therapeutic paradigm of dual targeting VEGF and PDGF for effectively treating FGF-2 off-target tumors. Nat. Commun..

